# Whole Slide Quantification of Stromal Lymphatic Vessel Distribution and Peritumoral Lymphatic Vessel Density in Early Invasive Cervical Cancer: A Method Description

**DOI:** 10.5402/2011/354861

**Published:** 2011-08-25

**Authors:** C. Balsat, S. Blacher, N. Signolle, A. Beliard, C. Munaut, F. Goffin, A. Noel, J. M. Foidart, F. Kridelka

**Affiliations:** ^1^Laboratory of Tumor and Development Biology, Groupe Interdisciplinaire de Génoprotéomique Appliquée (GIGA-Cancer), University of Liège, Pathology Tower (B23), 4000 Liège, Belgium; ^2^Department of Obstetrics and Gynecology, Hospital of la Citadelle, 4000 Liège, Belgium; ^3^Department of Obstetrics and Gynecology, CHU of Liège, 4000 Liège, Belgium

## Abstract

Peritumoral Lymphatic Vessel Density (LVD) is considered to be a predictive marker for the presence of lymph node metastases in cervical cancer. However, when LVD quantification relies on conventional optical microscopy and the hot spot technique, interobserver variability is significant and yields inconsistent conclusions. In this work, we describe an original method that applies computed image analysis to whole slide scanned tissue sections following immunohistochemical lymphatic vessel staining. This procedure allows to determine an objective LVD quantification as well as the lymphatic vessel distribution and its heterogeneity within the stroma surrounding the invasive tumor bundles. The proposed technique can be useful to better characterize lymphatic vessel interactions with tumor cells and could potentially impact on prognosis and therapeutic decisions.

## 1. Introduction

Metastases are responsible for more than 90% of all cancer deaths. In most carcinomas, lymph nodes are the first organs colonized by metastatic cells. The lymph node status (N+/N−) is amongst the strongest prognostic factors for overall survival and disease-free survival for patients with early cervical neoplasms [1, 2]. The exact mechanism by which tumor cells can metastasize away from the primary tumor is not fully understood but lymphatic vessels are viewed as the preferential route for tumor cell dissemination [3]. The increase in lymphatic microvessel number, due to the production by tumor cells of growth lymphangiogenic factors, could increase the probability that tumor cells enter in contact with lymphatic vessels and disseminate to regional lymph nodes [4]. In this context, the lymphatic vessel density (LVD) is proposed as a promising predictive marker of aggressive behavior and lymph node extension [5–9]. However, in the field of cervical cancer, studies on LVD yield inconsistent or contradictory results [10, 11]. 

To date, the LVD is evaluated using the method described by Weidner and colleagues [12] based on microvessel counting within preselected microscopic regions showing the highest neovascularization profile, called “hot spots”. These regions are thought to represent the areas of biological importance offering the highest probability for tumor cells to intravasate into lymphatics and disseminate away from the primary tumor. However, hot spot area assignment is reported to be subjective and observer dependant. Such selection biases are considered as the main reason for the lack of reproducibility among studies about the prognostic value of LVD [13, 14].

In order to overcome the limitations of the hot spot approach, two techniques allowing to inspect entire histological sections are proposed: the use of motorized stage scanning optical microscope or of autofocus device slide scanner. Both techniques are easy to use and are widely adapted for research [15]. Several studies have compared the hot spot method to that of whole slide high-resolution virtual image acquisition. These reports confirmed that the latter technique is more reproducible, easier to implement for standard quantification, and provides additional data adapted to the specific morphologies considered [14, 16, 17].

Digital image acquisition methods for analyzing, either hot spots or whole scanned sections, are sometimes accompanied by automatic or semiautomatic image analysis software. These software allows to process the image in order to extract vessels from the background and to perform measures such as vessel number and/or vessel size. However, in most cases, standard software is unable to accurately detect vascular structures and different teams have developed home-made image processing methods for specific applications [14, 16–18]. Their success depends critically on the immunohistochemical staining quality [19] which allows the discrimination of objects of interest by a coloration and/or a modification of grey-level intensities.

Our approach uses a high-resolution virtual imaging system coupled with image analysis tools. This methodology allows to determine, on one hand, an objective LVD quantification and, on the other hand, to detect possible spatial modifications of lymphatic vessel distribution resulting from tumor-stroma interactions. Different immunohistochemical techniques are first reviewed in order to illustrate the different features required for optimal automated detection. The image processing of slide scanning for analysis of lymphatic vessel segmentation in paraffin-embedded sections of cervical neoplasms is then described. Finally, objective LVD quantification and original measurements allowing quantifying the spatial distribution of lymphatic vessels surrounding the tumor bundles are presented. This technique is now available to give new insights into the tissue remodeling associated with cancer progression and vascular structure modifications.

## 2. Material and Methods

### 2.1. Tissue Collection and Processing

Surgical specimens of invasive cervical carcinomas were obtained from the biobank of the University of Liège (CHU, Liège Belgium) after study approval by the local ethics committee (CHU, Liège Belgium).

Immunohistochemical detection of podoplanin was carried out using an avidin-biotin-phosphatase assay on formalin-fixed paraffin embedded sections. Tissues were dewaxed in xylene and rehydrated through serial decreasing concentrations of ethanol/water solutions. Epitope retrieval was performed by heating slides in a target retrieval solution provided by the manufacturer (Dako S1699) (Dako, Heverlee, Belgium) during 11 minutes at 126°C at 1.4 bar of pressure. Solution was cooled at room temperature to avoid a fast temperature drop and nonspecific binding was prevented by incubation of 10% normal goat serum for 30 minutes. We used the primary monoclonal mouse antihuman antibody D2-40 that specifically recognizes the transmembrane mucin-type glycoprotein podoplanin mainly expressed by endothelial lymphatic cells [20] (Dako M3619, 1 : 100). It was applied during 90 minutes at room temperature. Subsequent reaction with second goat anti-mouse biotinylated antibody (Dako E0433) during 30 minutes was then achieved and tissue was finally incubated, during 30 minutes, with a complex of streptavidin and alkaline phosphatase (Jackson Immunoresearch, St Martens-Latem, Belgium, 1 : 2000). The phosphatase activity was revealed using the permanent red solution preliminary mixed with the endogenous phosphatase inhibitor levamisole (1 drop/mL). Tissues were finally counterstained with Carazzi hematoxylin 0.1% during 5 minutes.

### 2.2. Virtual Image Acquisition

Virtual images were acquired with the fully automated digital microscopy system dotSlide (Olympus, BX51TF, Aartselaar, Belgium) coupled with a Peltier-cooled high-resolution digital colour camera (1376 × 1032 pixels) (Olympus, XC10, Aartselaar, Belgium). Digital images of the whole tissue sections were digitized at high magnification (100x) producing virtual images in which pixel size is 0.65 *μ*m. It must be noticed that image processing was performed on original virtual image which size may exceed several gigabytes. Therefore, the time required for whole slide segmentation ranges approximately from 30 to 60 minutes with one processor of a computer equipped with Intel core i7 processor (2.80 GHz). Once the binary image was obtained, high image size hampers calculations. To overcome this limitation, before quantification, binary images were decimated according to the procedure previously described [18]. Image analysis was performed using image analysis library Pandore (GREYC, Caen France) and tool box of MATLAB software (9.2).

### 2.3. Lymphatic Vessel Density Quantification

LVD was defined as the number of lymphatic vessel section per mm² of stromal tissue which was automatically detected by a moment-preserving thresholding [21] applied on the blue component of the original image decimated 8 times. LVD was calculated for the entire stroma and more specifically for the peritumoral region located within 2 mm of tissue from the tumor invasion edge as it is proposed in the literature [5, 7].

## 3. Results and Discussion

### 3.1. Selection of Lymphatic Marker for Automated Detection

The discovery of several markers suitable to bring out the lymphatic endothelium has marked major advances in lymphangiogenesis study [22]. Most studies have mainly used vascular endothelial growth factor 3 (VEGFR-3), Prox-1, lymphatic endothelial hyaluronan receptor-1 (LYVE-1), and podoplanin to identify lymphatics [3]. However, these makers have their own features and are not fully comparable for lymphatic vessel detection. Therefore, for setting up an automated detection method, the initial selection of the best marker candidate is mandatory. 

The cell surface tyrosine kinase receptor VEGFR-3 mainly expressed by endothelial lymphatic cells can also be expressed by some fenestrated blood vessels [23] and then should not be used to discriminate lymphatics from blood vessels. Regarding Prox-1, a transcriptional factor driving specific lymphatic gene expression, its nuclear localization prevents it from being the ideal marker for quantifying lymphatic vessel microscopically [24]. Finally, LYVE-1 is reported to be downmodulated in response to inflammation [25] and its staining often underestimates the lymphatic vessel number when compared to D2-40 immunodetection in cervical cancer [11]. Based on these scientific evidences, the specific D2-40 antibody was selected for the present study. Nevertheless, although D2-40 shows high accuracy for lymphatic vessel detection, several limitations need to be noted: pluristratified epidermis is reported to be recognized by the D2-40 [26] and the expression of podoplanin can be induced in squamous cell carcinoma [10, 20].

### 3.2. Vessel and Tumor Detection

**Figure 1:**
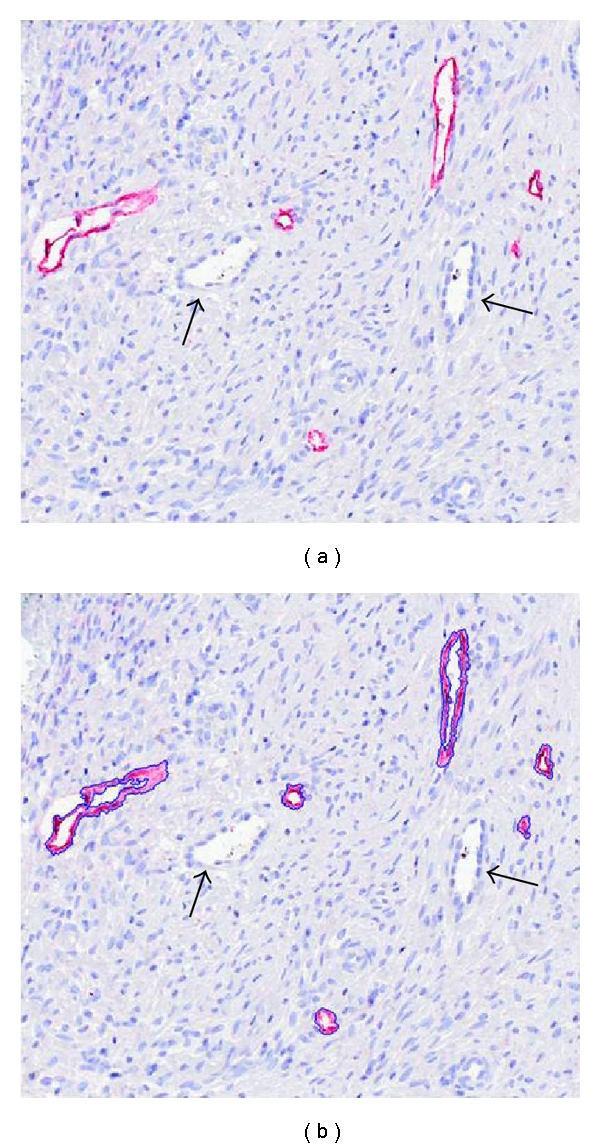
Immunohistochemical detection of lymphatic vessels. Lymphatic vessels were detected on a cervical cancer section by immunostaining with antipodoplanin antibody (D2/40) (red) (a). They are detected by automated segmentation (blue lines) (b). Blood vessels are not stained (arrows).

Figure 1(a) shows the detection of podoplanin after immunohistological staining using D2-40 antibody. The lymphatic vessels are specifically recognized and strongly stained in red with no background observed at the level of cervical cancer tissue. Such a staining allows an accurate detection of lymphatic vessels by automated segmentation (Figures 1(a) and 1(b)). However, given that structure detection is mainly based on color segmentation, weakly stained lymphatic vessels could remain unrecognized. Therefore, for automated detection, low specificity, weak staining, or high background must be avoided otherwise large detection mistakes can occur leading to erroneous measurements. In our experience, D2-40 antibody appears to be an appropriate specific marker whose detection provides an optimal contrast between lymphatic vessels and the tissue background. The segmentation processes described below, consisting in the transformation of the original image into a binary one, can then be performed automatically.

**Figure 2:**
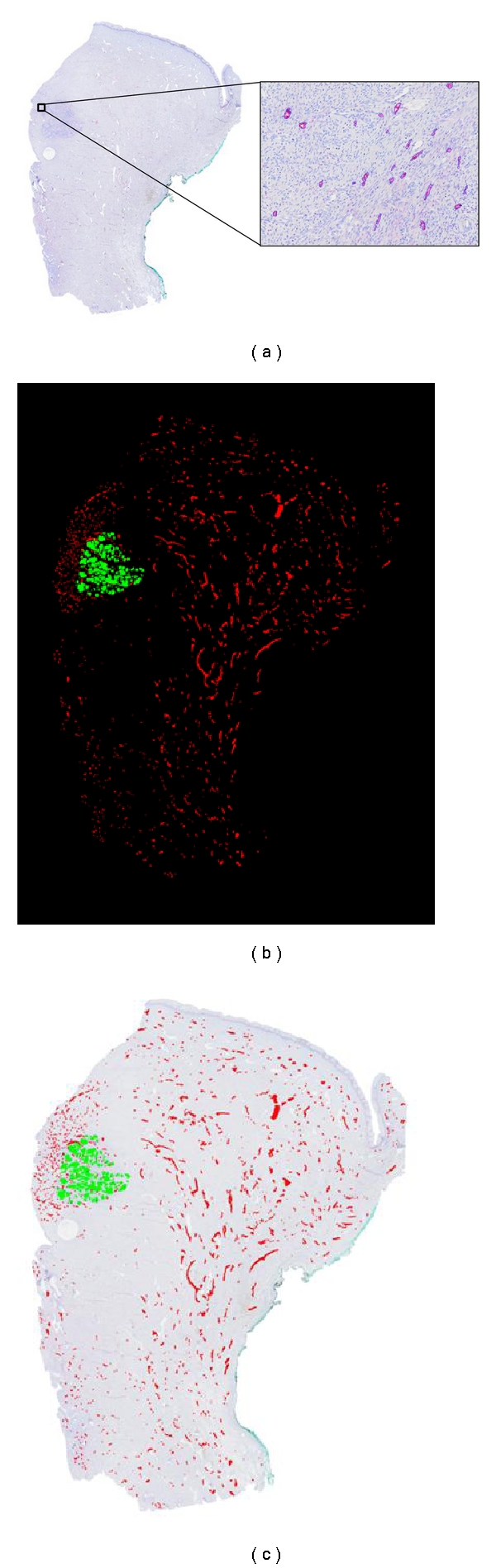
(a) Virtual image of whole cervical cancer section with lymphatic vessel detection (blue lines) achieved at high resolution (insert). (b) Binarized image of lymphatic vessels (D2/40 positive) (red) and tumor tissue (green). (c) Original image overlaid by binarized structures.

**Figure 3:**
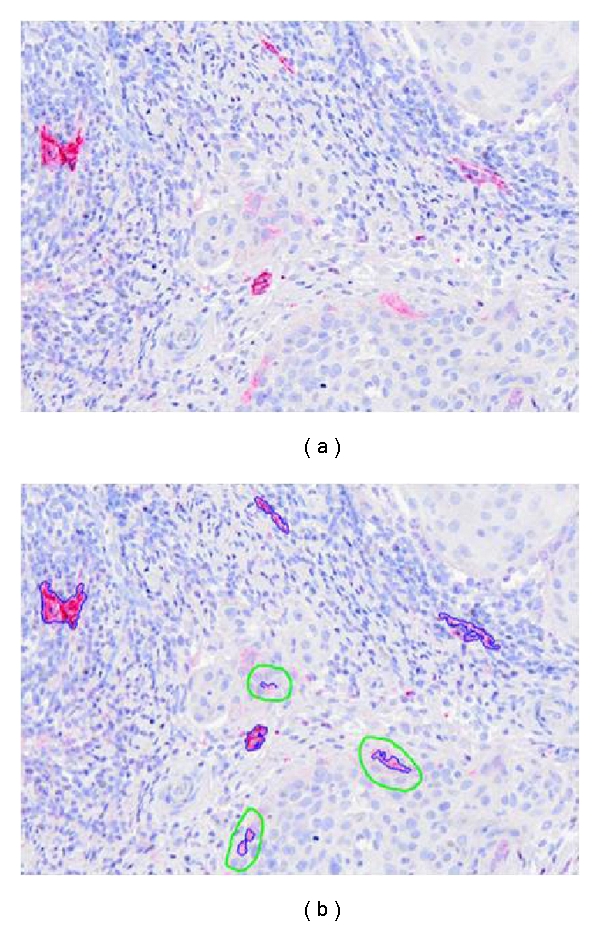
Immunohistochemical detection of lymphatic vessels. Vessel sections detected by using an antipodoplanin antibody appear in red (a) and are automatically segmented in blue (b) by image processing (blue lines). Some tumor cells positive for podoplanin are manually eliminated (green circles in b).

In digitized color images (RGB), stained lymphatic vessel walls appear in red (Figures 1 and 2). In order to increase the contrast between the vessel endothelium and the surrounding tissue, the excess red component (two times red value minus blue value minus green value) is calculated. The lymphatic vascular structures are then easily detected on the whole virtual slide using automatic entropy thresholding [27]. Additionally, to take into account the entire lymphatic vessels, lumens are identified as the lightest structures in the tissue (the value of the threshold depends on the average value of the background). To avoid the selection of other structures (i.e., blood vessel lumen, holes), only lumens neighboring the previously detected lymphatic walls are considered. Finally to avoid misinterpretation due to tumor and epidermal cells D2-40 recognition (see above), nonlymphatic detections are removed manually (Figure 3). 

To verify the accuracy of our methodology in detecting all lymphatic vessels identified by immunohistochemistry, manual count was performed on 5 distinct cervical cancer tissue sections, and results were compared with those obtained by automated vessel detection program. Results indicate that 93% ± 3.5 of lymphatic vessels are correctly detected and counted on the whole tissue using the automated method. 

Once lymphatic vessel sections are detected on the whole slide, the tumoral tissue is manually delineated on the virtual image. The manual delimitation is currently the easiest method to delineate the tumor bundles on tissue sections. However, the immunolabeling of cytokeratin could be used to identify carcinoma cells. In this case, the same procedure of segmentation to the one used for lymphatic structure would allow an automated detection of tumor cells.  Figure 2(b) illustrates the binarized image of vessels and tumor.

### 3.3. LVD Measurement

Once binary images are obtained, parameters such as tissue surface, lymphatic vessel number, and LVD can be measured in any part of the tissue using standard software. For illustration purposes, these parameters were assessed on the cervical cancer sample presented above (Figure 2). Results are presented in Table 1 and compared with those determined with hot spot technique.

In this particular example, the assessed peritumoral area and the number of lymphatic vessels are, respectively, more than 60 and 10 times higher than those determined with the hot spot approach. This result of the proposed measure shows that total microenvironment can be taken into account. This enables to provide an objective measurement which is not subjected to inter- and intraobserver variability.

### 3.4. Spatial Lymphatic Vessel Distribution

**Figure 4:**
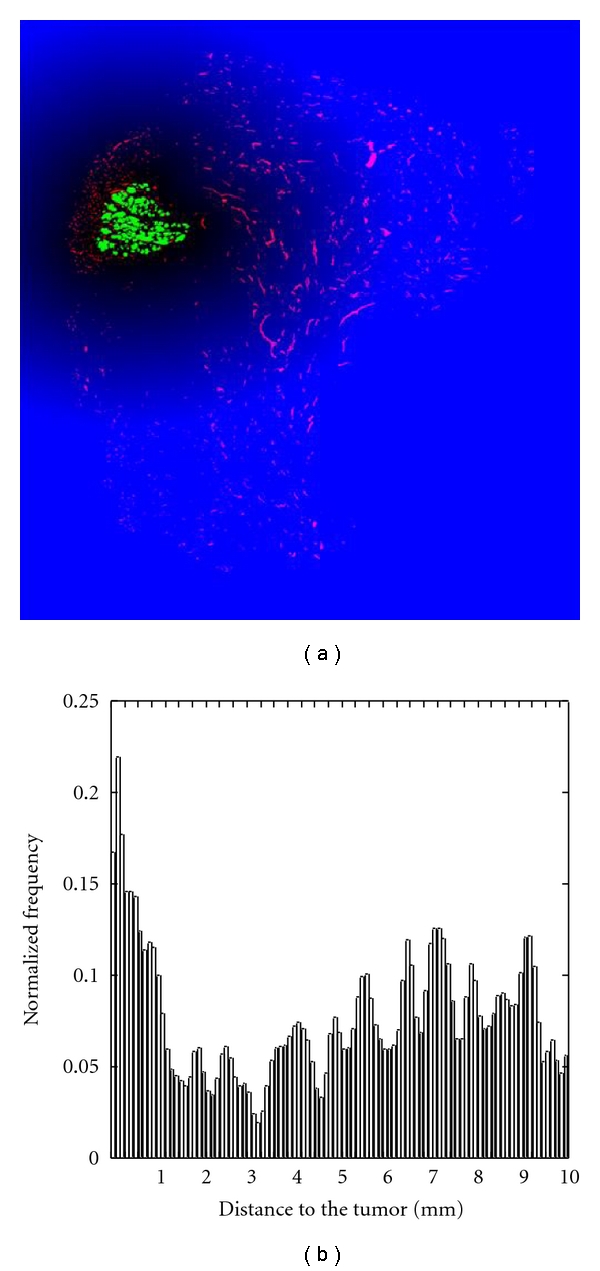
(a) Euclidean distance function applied to the binary image generated in Figure 2(b). The intensity of each pixel of the blue background indicates the distance of this pixel to the nearest point to the tumor. The more a pixel is far from the tumor, the more its intensity is bright. (b) Normalized histogram representing the number of vessels in function of their distance to the tumor.

The visual observation of both original and processed images (Figures 2(a) and 2(c)) shows that lymphatic vessels are not homogeneously distributed throughout the tissue. Although LVD value can inform us about level of lymphangiogenesis activity near the tumor, it does not allow to characterize the tumor heterogeneity. Because our main focus is to study the tumor-vessel interface, quantification is focused on the spatial distribution of vessels in relation to the tumor front of invasion. The position of all vessels in relation to tumor cells is determined. This is performed by applying the Euclidean distance function [28] to the complementary image of the binary image of the tumor. By this transformation, it is assigned to each pixel surrounding the tumor a color intensity corresponding to the distance between that pixel and the nearest point of the tumor. This is illustrated in Figure 4(a) in which the blue gradation of the background is proportional to the distance of each pixel to the nearest pixel belonging to the tumor mass. The value of the pixel corresponding to the centre of mass of each vessel gives a measure of the distance separating vessel from the tumor. From these data, a histogram representing the number of vessels in function of its distance to the tumor is generated (Figure 4(b)). In the representative example of Figure 4, the vessel distribution displays a bimodal pattern indicating the presence of two distinct areas. The first one corresponds to vessels clustered around tumor cells covering a radius of about 2-3 mm. The second area contains a large number of vessels scattered between this limit and the tissue border.

**Figure 5:**
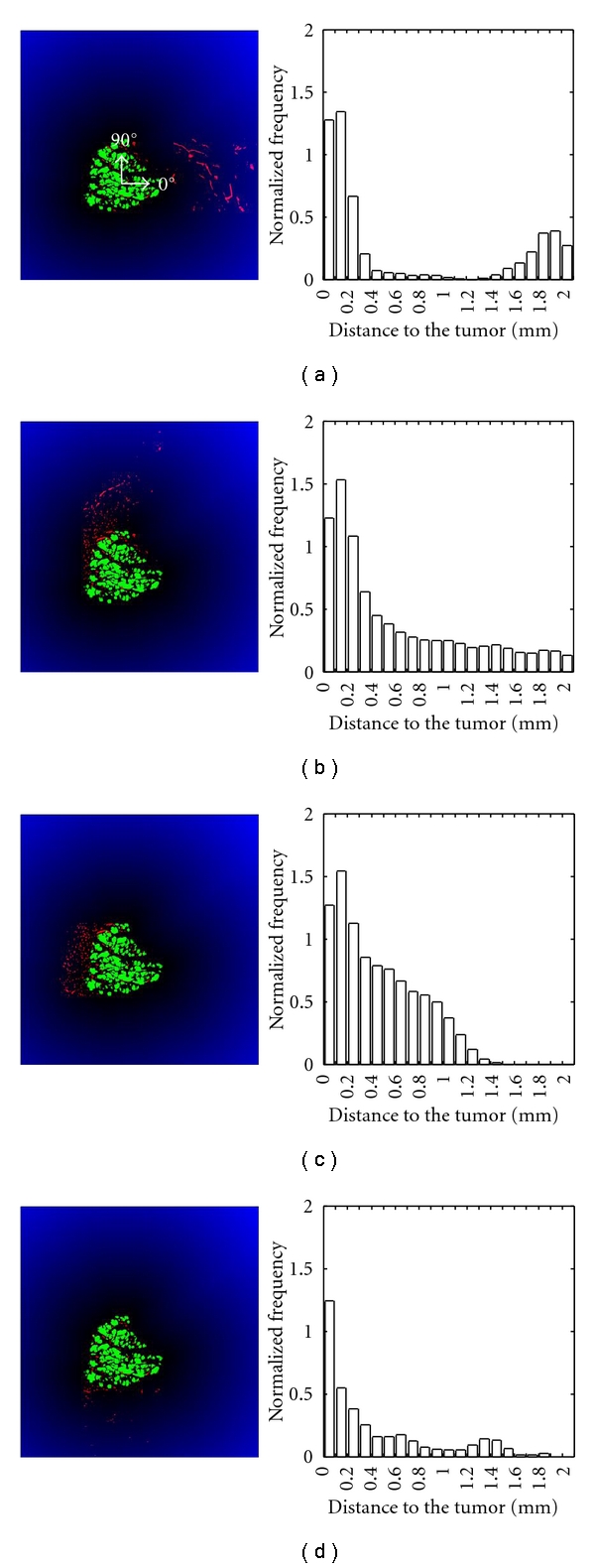
Euclidean distance function applied to the region of interest around the tumor. Vessels (in red) oriented at 0° (a), 90° (b), 180° (c) and 270° (d) are shown on the left (green = tumor cells). The histograms on the right correspond to the vessel distribution (normalized frequency) as a function of the distance to the closest tumor nodules (mm).

Once the region of interest (2-3 mm around the tumor) is determined, the degree of vessel distribution uniformity around the tumor is investigated. From the tumor mass centre, directional vessel distributions was calculated from 0° to 360° each 45° (counter clockwise). Images of vessels oriented at 0°, 90°, 180°, and 270° from the tumor and the corresponding histogram of distribution (normalized frequency) for distances between 0–2 mm (insert), are presented in Figure 5. A visual observation of images indicates that vessels cluster in the left part of the image. The spatial histogram distribution allows to objectively quantify the extent of this feature.

**Figure 6:**
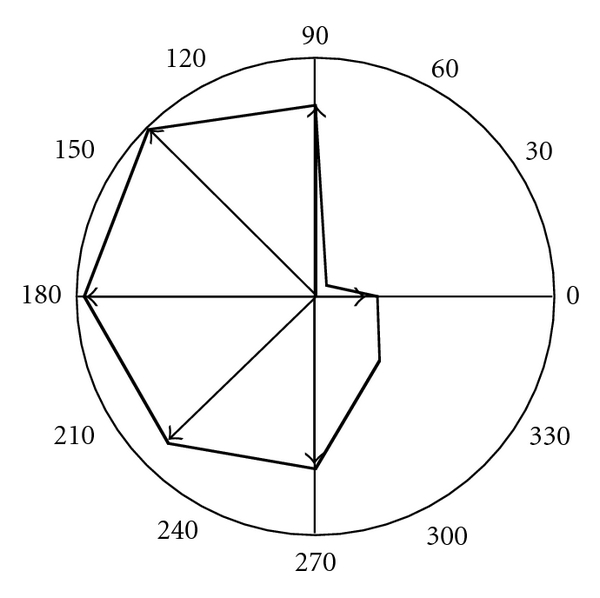
Polar representation of the density of vessels by unit of length measured from the tumor mass centre.

The area under the distribution histogram is a measure of the vessel density by unit of length. This vessel density is calculated for the 8 considered directions and drawn on a polar graph (Figure 6) with the goal of better-visualizing the vessel distribution around the tumor. The presence of lymphatics is clearly detected in the area ranging from 90° to 270° directions, underlying the heterogeneity of vessel distribution around the tumor.

## 4. Conclusion

Conventional optical microscopy technique (hot spot) applied to characterize angiogenesis has shown low reproducibility due to high interobserver variability [14, 16, 17]. To date, most studies conducted on cervical cancers have used the hot spot technique to assess the peritumoral lymphatic vessel density. Consequently, despite initial promising data, the predictive value of lymphatic vessel density remains unproven [6–11]. Thanks to the emergence of digital virtual microscopy, whole biological tissue samples can now be studied. This requires robust automated methods for quantification. In the present work, we provide a new method of vessel quantification that can be performed on whole tissue sections. To ensure the accuracy of the proposed measurements, two preliminary steps are mandatory: (i) the appropriate immunohistochemical staining with optimal contrast between background and the structures to be quantified and (ii) an optimal image processing. The novelty of this method relies on its capacity to assess the vessel distribution in the vicinity of tumor bundles to give new insights into phenomenon taking place at the tumor-lymphatic vessel interface. Presently, this proposed method is applied to cervical cancer samples to illustrate how the obtained information can contribute to a better understanding of lymphatic vessel interactions with tumor cells. Although clinical implications remain to be proven, this information is expected to be of most interest for diagnosis/prognosis and for a better guidance of therapeutical decisions. This technique can also be applied to characterize lymphatic as well as blood vessel distribution in relation to any structure of interest.

## Figures and Tables

**Table 1:** LVD quantification in the whole cervical cancer tissue and peritumoral area assessed by the automated lymphatic vessel detection method in comparison with hot spot method described by Weidner and colleagues [12].

	Whole tissue	Peritumoral area	Hot spot
Tissue surface (mm^2^)	294.91	38.21	0.6
Vessel section number	2084	463	43
LVD (vessel number/mm^2^)	7.07	12.12	76.67
